# Annotations

**Published:** 1902-11-08

**Authors:** 


### ANNOTATIONS.

### The Pathology of Minor Ailments.

In the ceaseless search to discover the causes of life's great ills and unmask the agencies making for disease and death, comparatively little attention is given to the investigation of the pathology of life's little ailments. The slight sicknesses, the passing aches and pains, the little distresses are often fraught with issues of the most momentous character. It is frequently the trivial wrong that unbars the door to a serious invasion; the insignificant wound affords ingress to the death-dealing microbe; the trifling derangement gives place to permanent disorganisation. It is the little rift within the lute that in only too many cases makes mute life's music. In the unravelling of the mysteries of minor ailments, the practitioner approaching his task in a scientific spirit becomes one with the serious students of research. It is often thought that the puzzles of Nature are only to be solved in elaborate and expensively equipped laboratories; and that the unveiling of natural law might only be undertaken by the high priests of science. And yet in the past some of the most noteworthy discoveries have been made by quiet and secluded workers, and some of the most beneficent reforms have been introduced by country practitioners. In the unfolding of the causation and nature of the pathological processes underlying those conditions we are wont to term minor ailments, the general practitioner has opportunities rich and almost boundless.

### The Hygiene of Burial.

Our little island is congested with the living and overcrowded by the dead. The coming and going of the human slowly and silently re-inhabitates the land. The mysteries of birth and death lie hidden in the great insoluble problem of life. We put our dead out of sight, and try to forget the dissolution and decay which necessarily overwhelms organic matter when the breath of life has fled. Funeral customs are exerting a tyranny from which serious minds would gladly escape. Methods of burial now adopted only too frequently offer opportunities for the spread of disease. The dead are in many instances a serious menace to the living. We have much sympathy with all rational efforts to secure reform in our burial procedures. The Church of England Burial Reform Association has recently held its annual meeting, and during its twenty-four years of quiet influence has accomplished much good. It has done much to secure a saner and more sanitary method of dealing with the dead. There is still much need for wiser and more scientific action. Every necessary precaution should be adopted to protect the interests of the living. There is need for timely, instead of delayed, burial. Coffins of *papier maché*, or wicker, or some such material as will perish in suitable soil as soon as the body itself, should be used. Although we are strongly of opinion that cremation affords the best method for dealing with all infectious bodies, yet much improvement might be made in securing proper sanitary burial in earth sufficient for the purpose, and of a nature to effect the dissolution of the body. In all funeral arrangements it should be realised that simplicity is most seemly. The hygiene of burial is not a popular subject, but it should be faced by all serious and thoughtful minds; and it would be well if men and women, who have worked for the safety of their fellows during their life, made adequate arrangements that when they shuffle off this mortal coil the useless rags of humanity should not be allowed to become instrumental for evil.

### The Rejuvenescence of the Manchester Infirmary.

As we indicated recently, Manchester men have at last forced action in regard to the rebuilding of the ancient city hospital. The so-called "Board of Management" have realised that the process of evolution even embraces "effecte degentavies" and in a dying effort they have unanimously adopted the following "in memoriam": "That the whole Board of Management as now constituted resign and vacate office on November 13th next, and that a special general meeting of trustees be held in the board room of the infirmary on Friday, November 14th next, at 11 A.M., for the election of a new board of management." It would be churlish to speak ill of the dying board, but we sincerely hope "the coming race" will not perpetuate the temper and methods which for so many years have blocked hospital reform in Manchester.

### The Public-House and the Spread of Consumption.

The abuse of alcohol furnishes subject for endless discussion. The evils of intemperance stand apparent to the most casual observer. All sorts and conditions of men are interested in the mitigation of the curse. The "Drink Problem" furnishes material peculiarly attractive to the politician, the sociologist, the humanitarian, the sanitarian and the pathologist. Of recent years alcoholism in its various manifestations has received serious study, and much new light has been thrown on the subject. As evidence of the importance of the matter, as viewed from the purely medical standpoint, we need only draw attention to the fact that the November issue of *The Practitioner* constitutes a special number dealing with different phases of the Alcohol Question. An important aspect of the subject is dealt with by Dr. Kelynack in the current number of *The British Sanitarian.* Evidence is there collected from most reliable sources to show that many public-houses are seriously lacking in sanitary requirements and furnish conditions peculiarly suitable to the propagation of pulmonary tuberculosis. It has been conclusively shown that the alcoholic subject is predisposed to tubercular infection, and now it would seem clear that tuberculous dust is constantly present on the floor or in the air of many a public house; and there is no denying the fact that advanced phthisical cases often congregate in these places. The matter is receiving the careful attention of medical officers of health, and it will be well that public opinion should insist on the adoption immediately of effective hygienic measures in all public-houses.

